# Textural analysis and artificial intelligence as decision support tools in the diagnosis of multiple sclerosis – a systematic review

**DOI:** 10.3389/fnins.2024.1457420

**Published:** 2025-01-21

**Authors:** Filip Orzan, Ştefania D. Iancu, Laura Dioşan, Zoltán Bálint

**Affiliations:** ^1^Department of Biomedical Physics, Faculty of Physics, Babeş-Bolyai University, Cluj-Napoca, Romania; ^2^Faculty of Mathematics and Computer Science, Babeş-Bolyai University, Cluj-Napoca, Romania

**Keywords:** multiple sclerosis, MRI, artificial intelligence, computer assisted diagnosis, U-Net, radiomics, textural analysis

## Abstract

**Introduction:**

Magnetic resonance imaging (MRI) is conventionally used for the detection and diagnosis of multiple sclerosis (MS), often complemented by lumbar puncture—a highly invasive method—to validate the diagnosis. Additionally, MRI is periodically repeated to monitor disease progression and treatment efficacy. Recent research has focused on the application of artificial intelligence (AI) and radiomics in medical image processing, diagnosis, and treatment planning.

**Methods:**

A review of the current literature was conducted, analyzing the use of AI models and texture analysis for MS lesion segmentation and classification. The study emphasizes common models, including U-Net, Support Vector Machine, Random Forest, and *K*-Nearest Neighbors, alongside their evaluation metrics.

**Results:**

The analysis revealed a fragmented research landscape, with significant variation in model architectures and performance. Evaluation metrics such as Accuracy, Dice score, and Sensitivity are commonly employed, with some models demonstrating robustness across multi-center datasets. However, most studies lack validation in clinical scenarios.

**Discussion:**

The absence of consensus on the optimal model for MS lesion segmentation highlights the need for standardized methodologies and clinical validation. Future research should prioritize clinical trials to establish the real-world applicability of AI-driven decision support tools. This review provides a comprehensive overview of contemporary advancements in AI and radiomics for analyzing and monitoring emerging MS lesions in MRI.

## 1 Introduction

Multiple sclerosis (MS) is an autoimmune disease of the central nervous system (CNS) that is manifested by the presence of demyelinated areas in the CNS (Kuhlmann et al., 2017). This disease affects approximately 2.8 million people globally, with a higher incidence in women aged 20–50 years (Wijeratne and Carroll, 2021). According to 2017 McDonald criteria (Thompson et al., 2018), diagnosis of MS combines clinical, imaging, and laboratory evidence. Neurological examination is combined with imaging [magnetic resonance imaging (MRI) or optical coherence tomography] and neurophysiological testing (visual evoked potentials). In patients who have clinical symptoms and lesions on MRI, cerebrospinal fluid is collected through lumbar puncture. The presence of oligoclonal bands in cerebrospinal fluid confirms the diagnosis of MS (Thompson et al., 2018).

**FIGURE 1:**
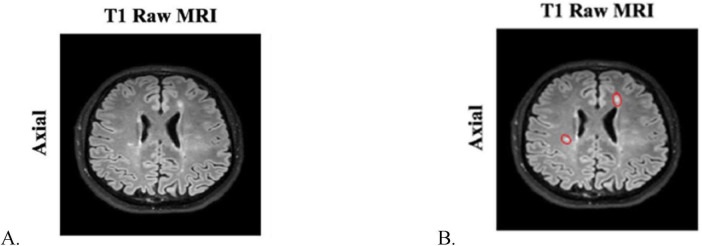
**(A)** Axial T1 MRI image of a person with MS lesions; **(B)** manually annotated MS lesions in an axial T1 MRI image (Sarica and Seker, 2022).

Magnetic resonance imaging techniques such as double inversion recovery, phase-sensitive inversion recovery, and magnetization-prepared rapid acquisition with gradient echo sequences are used to highlight MS lesions of cerebral cortex. These regions are areas of hyperintense white matter present in MRI images acquired by the T1, T2, or fluid attenuated inversion recovery (FLAIR) method (Hitziger et al., 2022). On Figure 1A, there is an example MRI T1 image with two lesions which appear as hyperintense areas of white matter (Sarica and Seker, 2022). An area of hyperintensity which has at least 3 mm in long axis is considered a lesion (Thompson et al., 2018). The monitoring of the evolution of the disease, but also the efficiency of the treatment is analyzed by the appearance or absence of new lesions on the yearly follow-up MRI images (Martínez-Heras et al., 2023).

Manual identification and delimitation of demyelinated areas on MRI images (Figure 1B) has some drawbacks, being time-consuming and requiring qualified personnel. Whereas its results depend on the experience of the expert interpreting MRI images. In addition to the subjectivity of the human factor, differences may occur due to MRI images acquired at different resolutions or with various qualities. To reduce these shortcomings, several automatic solutions for diagnosing and monitoring MS have been proposed (Shoeibi et al., 2021). Results comparable to expert annotation were obtained by using neural networks in deep learning algorithms combined with textural analysis (Commowick et al., 2021a).

Textural analysis is a known and promising technique in medical image processing with notable results in detecting sclerotic lesions (Elahi et al., 2020; Boca et al., 2023). Usually, attempts are made to detect lesions through those characteristics that are image invariant to variations in intensity, lighting, geometric transformations, or noise. For this the interrelationships of pixel intensities and pixel distribution are quantified, thus, obtaining numerous features. These features can be divided into the following categories: first-order features (gray-level histogram analysis), second-order features (gray-level dependence matrices), spectral features, and fractal features (wavelet transform and Fourier transform). The pixels identified with random texture are categorized as noise (Friconnet, 2021). To improve the signal-to-noise ratio and to reduce noise, preprocessing operations consisting of mathematical filtering are applied to the MRI images. As an example, Gaussian bandpass filters are used to remove the background noise (Kumar et al., 2023).

Due to the appearance of automatic methods for detecting lesions in medical images (Lambin et al., 2012), it was necessary to develop a methodology to analyze and evaluate the reproducibility and quality of results by automatic detection methods. Radiomics has been gradually applied to the analysis of pathological damage, diagnosis, differential diagnosis, and prognosis of MS. Machine learning (ML) models that use radiomics features are developed to detect MS lesions (Peng et al., 2021). The methods of radiomics consists of converting medical images into mineable data via the extraction of various quantitative imaging features (Lu et al., 2019).

The purpose of this article is to provide a review that presents the current state of use of artificial intelligence (AI) and ML in diagnosing and monitoring MS. The aim of our work was to explore how MS is diagnosed and monitored using AI/ML methods applied on MRI images and whether the texture features of these imagining modality are considered. The motivation that drives us to conduct this systematic literature review (SLR) is given by the following reasons:

1. To characterize the state-of-the-art to identify and understand the ongoing scientific research on MS identification; and
2. To position our future work in the current research.

To address the goal of our SLR the following research question was defined: *What are the most effective machine learning algorithms for diagnosing multiple sclerosis?*

## 2 Materials and methods

### 2.1 Literature research and study selection

For this review article, we used PRISMA principle to perform an objective search of publications investigating MRI-based radiomics applications to MS without time constraint. The following key terms were used: “multiple sclerosis” AND “magnetic resonance imaging” AND “Neural Network” AND (“radiomics” OR “texture analysis”) AND (“AI” OR “Artificial intelligence” OR “Machine Learning”).

Based on the above-mentioned criteria, we selected the publications that: (1) evaluated MS or other brain damage using an MRI-based radiomics approach; (2) had human participants and (3) were written in English.

Exclusion criteria included the following: (1) studies based on other imaging modalities, e.g., ultrasound, CT, and PET-CT; (2) publications designed as letters to the editor, editorial, conference abstract, and review; (3) were performed on animals; (4) did not use AI or ML.

**FIGURE 2:**
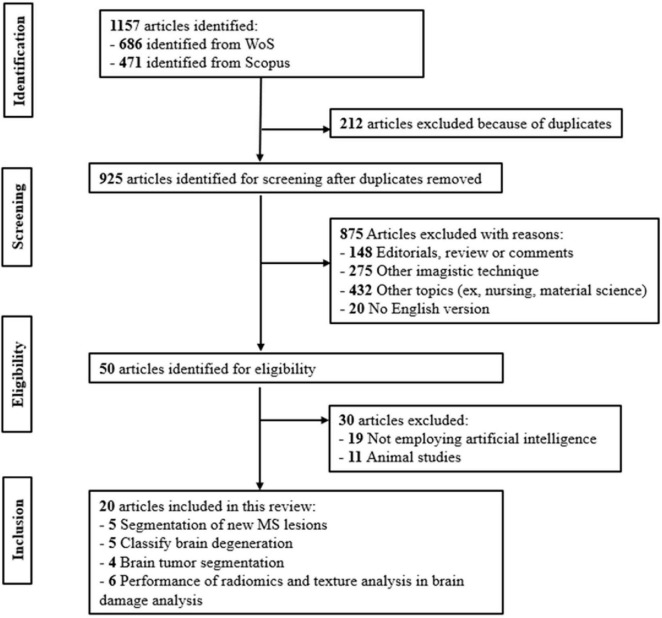
PRISMA flowchart.

We first obtained a number of 1,157 articles from WOS and Scopus, whereas at the end there were only 20 articles which satisfied all the criteria (8 articles from Scopus and 12 articles from WOS). The articles were initially independently retrieved from both databases, followed by an exclusion of the duplicates. Additionally, studies focusing on imaging techniques outside the scope of our research, as well as non-English articles, were excluded. In the final step of the selection, 50 articles met the eligibility criteria, and after careful handpicking based on their relevance to the topic under investigation, only 20 remained eligible (Figure 2).

### 2.2 Data extraction

To have a better overview, we searched and extracted manually the general data and put them in a pre-defined table with:

1. General features, including the name of authors, publication year, and journal;
2. Study characteristics, including general aim, sample size, study design (prospective and retrospective), and MRI technical data (e.g., type of scanner, field of strength, and sequences used for radiomics analysis); and
3. Details of radiomics analysis including image preprocessing, segmentation method, software used for segmentation and feature extraction, number and type of extracted features, feature selection methods/machine learning classifiers, and number of radiomics features used.

## 3 Results

### 3.1 Data collection methods used in the included studies

Using the above-mentioned keywords, we retrieved a number of 1,157 articles from WOS and Scopus. After successively filtering them according to the above-mentioned criteria, we obtained 20 articles which were included in this review. These articles are from the timespan: 2015–2023, with 60% (12 out of 20) of them from the last 2 years. On Figure 2 the PRISMA diagram used to filter the relevant articles is presented. It shows the sorting process and explains the exclusion criteria applied. Exclusion criteria included the following:

1. Studies based on other imaging modalities, e.g., ultrasound, CT, and PET-CT;
2. Publication types as letters to the editor, editorials, conference abstracts, or review papers;
3. Studies which were performed on animals;
4. Or which did not use AI or ML.

As shown in Table 1, the 20 articles were published in 18 distinct scientific journals. Notably, only two journals—Computerized Medical Imaging and Graphics and Frontiers in Neuroscience—had two articles, highlighting the diverse range of publications included in the analysis.

**TABLE 1:** General characteristics of included studies.

Reference	Periodical	Study design	No. of patients (train vs. test cohort)	Analyzed outcome	MRI sequence	Readers (no. of radiologist)	Scanner
Jain et al., 2021	Hindawi Computational and Mathematical Methods in Medicine	Prospective	1,374	Classification of brain degeneration	T1 and T2	1	3T Siemens TIM Trio, 3T Siemens Magnetom Vida, 1.5T Siemens Sonata, 1.5T Siemens Avanto
Cuocolo et al., 2020	Neuroradiology	Retrospective	89 (71:18)	Classification of adenomas	T2W	1	1.5T Philips; 3T Siemens
Demirel et al., 2021	Èeská a Slovenská Neurologie a Neurochirurgie	Retrospective	60	Classification of brain metastasis	T1W	1	1.5T Philips Intera and 1.5T Siemens Aera
Wang et al., 2023	European Journal of Radiology	Retrospective	210 (147:63)	Diagnostic performance of a hippocampal radiomics models	T2-FLAIR	2	3T Siemens Magnetom Trio Tim and Magnetom Skyra
Zhong et al., 2017	Brain Imaging and Behavior	Prospective	72	Regional gray matter measures, classification of MS participants	T1W	1	3T Siemens Magnetom Trio Tim
Zhou et al., 2022	Hindawi International Journal of Clinical Practice	Retrospective	114 (80:34)	Tumor grade	T1WI	1	3T GE Discovery MR750 W
Kumar et al., 2023	Journal of Personalized Medicine	Retrospective	83	Tumor grade	T2W	2	1.5T Phillips; 3T General Electric
Lu et al., 2019	European Radiology	Retrospective	152 (106:46)	Classification of meningioma	T1W, T2W, and T2-FLAIR	2	3T Verio Siemens, 3T DISCOVERY MR750W, GE
Elahi et al., 2020	Computerized Medical Imaging and Graphics	Retrospective	700 (490:210)	Detection of amyotrophic lateral sclerosis	T1W	1	3T Siemens Prisma, Siemens Trio, General Electric MR750
Dastmalchian et al., 2021	European Journal of Nuclear Medicine and Molecular Imaging	Retrospective	31	Differentiation between intra-axial adult brain tumors	T1 and T2	2	3T Verio and Magnetom Skyra; Siemens
Ortiz-Ramón et al., 2020	Physica Medica	Retrospective	100 (80:20)	Classification between glioma and brain metastasis	T1W	1	1.5 T Philips Achieva
Ortiz-Ramón et al., 2019	Computerized Medical Imaging and Graphics	Prospective	200	Presence/absence of a stroke	T1W, T2W, and FLAIR	1	1.5T GE Signa LX
Eshaghi et al., 2021	Nature Communications	Retrospective	9,390 (6,322:3,068)	Classification of MS subtypes	T1W, T2W, and FLAIR	1	3T and 7T
Combès et al., 2021	Frontiers in Medicine	Retrospective	95	Segmentation of new lesions	T1W, T2W, and T2-FLAIR	3	3T Siemens Magnetom Verio and 3 T Siemens VB17
Lefkovits and Lefkovits, 2022	Acta Universitatis Sapientiae, Informatica	Retrospective	369 (303:66)	Segmentation of brain tumors	T1, T1ce, T2, and FLAIR	2	3T Siemens Magnetom Verio
Fenneteau et al., 2021	Journal of Medical Imaging	Retrospective	51 (45:6)	Segmentation of MS lesions	FLAIR	4	1.5T and 3T
Sarica and Seker, 2022	Frontiers in Neuroscience	Retrospective	100 (60:40)	Detection of new MS lesions	T2 and FLAIR	4	1.5T and 3T
Ashtari et al., 2022	Frontiers in Neuroscience	Retrospective	100 (40:60)	Segmentation of new lesions	FLAIR	4	1.5T and 3T
Friconnet, 2021	Chinese Journal of Academic Radiology	Retrospective	300	Correlation of texture analysis features with brain area	T1W, T2W, and diffusion-weighted slices	2	3T Siemens Connectom
Pardini et al., 2015	American Academy of Neurology	Retrospective	93 (71:22)	Motor network integrity	T2	1	3T Philips Healthcare

Out of the 20 articles received, only two were prospective studies, indicating that the use of AI in diagnosing MS is still evolving and additional research and testing are necessary before it can be deemed suitable for clinical implementation.

The sample size is a crucial factor for reliable AI and machine learning (ML) analysis. Altogether the 20 articles included in the review sum up a total of around 18,000 patient datasets. The number of patients varied between 31 and 9,390, with a median of 100 and interquartile range (IQR) of 229.75 [−257; 661.8]. However, most published articles address the challenge of analyzing enough samples, with the majority reporting less than 100 patients during the training phase. The MRI sequences analyzed varied between studies, thus, making it difficult to compare the classification and segmentation results across different research.

T1, T2, and FLAIR sequences of the MRI acquisition protocols were used to extract the radiomics characteristics. Five articles out of 20 used only T1 and another 4 articles used only T2. The rest of the studies used images acquired through the combined sequences to extract the characteristic features. All the identified 20 articles provided information about the intensity of the MRI field upon acquisition. In 3 articles the field strength was 1.5T, whereas in 10 articles a 3T field and in 1 article a 7T field was used. Six articles report a combination of these two magnetic field strengths. It should be noted that in 13 articles 3D images were used for the analysis.

Some articles focus solely on addressing basic classification tasks, such as assigning a single label to an MRI scan, while others tackle more complex challenges, performing classification at the pixel or voxel level, e.g., image segmentation.

In 12 of the reviewed articles, the process for achieving ground truth segmentation was explicitly described. In two studies, annotations were manually performed by an expert with 20 years of experience. In five other studies, two experts, either neuro-oncologists or neuroradiologists, independently annotated the MRI images, and their results were compared. Any discrepancies between the annotations were reanalyzed and resolved through consensus. One article involved three experts: a senior expert with 10 years of experience annotated all images, while two experts with 5 years of experience independently reviewed the annotations for comparison. In four studies, annotations were performed by a panel of four or more experts using a voting system, with a senior expert holding veto power. Various tools were used for segmentation and annotation across the studies, including ITK-Snap, TextRad, 3D Slicer, MATLAB, and custom-built software.

The number of radiomic features analyzed across the studies ranged from 6 to 3,655, with a median of 156. After feature extraction, only stable features were retained, while those with low variance were discarded. Additionally, to improve robustness, features influenced by MRI noise and imaging heterogeneity were excluded (Boca et al., 2023). Statistical analysis of these features was conducted using R or MATLAB software.

**FIGURE 3:**
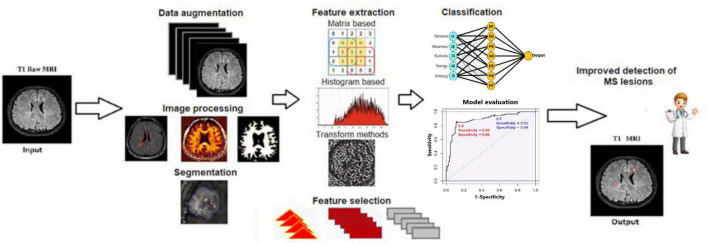
General diagram of the workflow (Wang et al., 2023; Zhou et al., 2022).

Eleven articles focused on classifying specific regions within the imaging data as their output. Among ML classifiers, Support Vector Machines (SVMs) and Random Forest (RF) were the most used, appearing in seven and six studies, respectively. Six articles focused on segmentation tasks, with five of them employing variations of the U-Net model. In eight studies, at least two different ML algorithms were compared. There was no fixed sequence of steps followed across the articles. Data augmentation techniques were applied in 6 of the 20 studies, and 9 studies involved image resampling. Similarly, image normalization was performed in 12 studies. A summary of the data can be found in Tables 1, 2. As shown in Table 2, depending on the workflow selected and developed by the authors, certain steps were omitted (marked with X). For a clearer representation of the key steps involved in detecting MS lesions, Figure 3 illustrates the primary stages of the process.

**TABLE 2:** General steps (X for absence of step and green dot for its presence).

Article	ML task	Input data	Mixing data	Data augmenta -tion	Image resamp -ling	Image normali -zation	Image pre-processing filters	Manual segmenta -tion	Automatic segmenta -tion	Texture analysis	Feature extrac -tion	Feature selection	Feature statistical analysis	Test multiple classifier
Jain et al., 2021	Classification of brain degeneration	3D MRI images				**X**		**X**						**X**
Cuocolo et al., 2020	Classification of adenomas	3D MRI images	**X**	**X**					**X**				**X**	**X**
Demirel et al., 2021	Classification of brain metastasis	2D MRI images	**X**	**X**			**X**	**X**						
Wang et al., 2023	Binary classification	3D MRI images	**X**	**X**		**X**		**X**						**X**
Zhong et al., 2017	Binary classification	3D MRI images and behavior tests	**X**	**X**	**X**	**X**			**X**	**X**				**X**
Zhou et al., 2022	Binary classification	3D MRI images and pathological tests		**X**	**X**	**X**	**X**	**X**						**X**
Kumar et al., 2023	Classification	2D MRI images and histological confirmation	**X**	**X**					**X**				**X**	
Lu et al., 2019	Classification of meningioma	2D MRI images		**X**	**X**				**X**					
Elahi et al., 2020	Classification	2D MRI images			**X**				**X**				**X**	
Dastmalchian et al., 2021	Classification	2D MRI images		**X**	**X**	**X**	**X**		**X**					
Ortiz-Ramón et al., 2020	Classification between glioma and brain metastasis	2D MRI images		**X**	**X**		**X**			**X**				
Ortiz-Ramón et al., 2019	Classification	3D MRI images	**X**	**X**	**X**	**X**	**X**	**X**						
Eshaghi et al., 2021	Classification of MS subtypes	2D and 3D MRI images		**X**	**X**		**X**	**X**		**X**				**X**
Combès et al., 2021	Segmentation of new lesions	3D MRI images	**X**			**X**		**X**		**X**	**X**	**X**		**X**
Lefkovits and Lefkovits, 2022	Segmentation of brain tumors	3D MRI images	**X**						**X**	**X**	**X**	**X**	**X**	
Fenneteau et al., 2021	Segmentation of MS lesions	3D MRI images	**X**	**X**				**X**		**X**	**X**	**X**	**X**	**X**
Sarica and Seker, 2022	Detection of new MS lesions	3D MRI images			**X**			**X**		**X**			**X**	**X**
Ashtari et al., 2022	Segmentation of new lesions	3D MRI images							**X**	**X**	**X**	**X**	**X**	**X**
Friconnet, 2021	Correlation of texture analysis features with brain area	2D MRI images		**X**	**X**	**X**	**X**	**X**						**X**
Pardini et al., 2015	Prediction	3D MRI images	**X**	**X**	**X**				**X**	**X**	**X**	**X**		**X**

Elahi et al. (2020) effectively emphasize the significant influence of various preprocessing steps on the final model accuracy, illustrating how these steps can markedly alter the model’s predictive performance. Their work demonstrates the importance of careful consideration and optimization of preprocessing techniques to enhance the reliability and precision of ML models in medical image analysis. The author achieved an 8.80% increase in model accuracy by applying their proposed normalization method, compared to cases without normalization. Also, they observed that downsampling the original MRI images to lower resolutions significantly improves classification accuracy. Their findings suggest that this adjustment in image resolution can substantially enhance the performance of machine learning models in differentiating between various conditions in medical image analysis. Their study analyzed classification accuracy across three downsampled MR image resolutions (2, 3, and 4 mm) compared to the original 1 mm images, achieving improvements of 3%, 6%, and 2%, respectively. This highlights the impact of image resolution on enhancing model accuracy. They also prove that using an ensemble model of classifiers for classification outperforms the best single linear classifier by a significant margin up to 2%.

### 3.2 Performance of the included studies

As shown in Table 3, various performance evaluation metrics were employed depending on the model’s output. Accuracy was the most used metric, appearing in nine articles, while the Dice score was used in four, whereas the area under the curve (AUC) was used in three. Other evaluation metrics included sensitivity, connectivity matrix, intraclass correlation coefficient (Koo and Li, 2016), Pearson’s correlation coefficient (Blyth, 1994), and the concordance index (Longato et al., 2020).

**TABLE 3:** Performance of AI models across included articles.

Article	AI technique	AI output	Performance
Jain et al., 2021	XGBoost classifiers	HT/DT	Accuracy: 97.38%
Cuocolo et al., 2020	Extra Trees classifier	ST/FT	Accuracy: 93.00%
Demirel et al., 2021	ANN, SVM, LR, RF, and NB	M/GBM	ANN Accuracy: 91.00%
Wang et al., 2023	SVM	HT/DT	Accuracy: 86.30%
Zhong et al., 2017	SVM	HT/MP/MI	Accuracy: 85.61%
Zhou et al., 2022	LR	LGG/HGG	Accuracy: 84.80%
Kumar et al., 2023	RF, SVM, GB, NB, and AB	LGG/HGG	RF Accuracy: 83.00%
Lu et al., 2019	DT, CIT, and DF	GI, GII, GIII	DF Accuracy: 79.51%
Elahi et al., 2020	Stacking of multiple classifiers! Meta-classifier: SVM (linear), SVM (RBF), KNN, FCNN (3 layers), and RF	HT/DT	Accuracy: 65.60%
Dastmalchian et al., 2021	Hypothesis tests	LGG/HGG	Area under the curve: 0.95
Ortiz-Ramón et al., 2020	RF, SVM, KNN, NB, and MLP	M/GBM	MLP area under the curve: 0.91
Ortiz-Ramón et al., 2019	SVM and RF	HT/DT	SVM area under the curve: 0.77
Eshaghi et al., 2021	Machine learning algorithm: SubStaIn	HT/PPMS/SPMS/RRMS	Concordance index: 0.63
Combès et al., 2021	nn-UNet	HT/DT	Sensitivity: 0.90
Lefkovits and Lefkovits, 2022	UNet, VGG16-UNet, and ResNet-UNet	WT/ET/TC	Ensemble model Dice score: 0.87
Fenneteau et al., 2021	Minimally Parameterized UNet	HT/DT	Dice score: 65.00%
Sarica and Seker, 2022	UNet with Attention Gate	HT/DT/WM	Dice score: 58.70%
Ashtari et al., 2022	Pre-UNet encoder-decoder	HT/DT	Dice score: 40.30%
Friconnet, 2021	Hypothesis tests	Correlate TA feature with semantic feature: repetitiveness, linearity, directionality, roughness, randomness, coarseness	Intraclass correlation coefficient, Pearson’s coefficient correlation
Pardini et al., 2015	Spearman and Pearson correlation	Parameter correlation: EDSS scores for MS	Connectivity matrix

HT, healthy tissue; DT, diseased tissue; ST, soft tissue; FT, fibrous tissue; M, metastases; GBM, glioblastoma; MP, motor function preserved; MI, motor function impaired; LGG, low-grade gliomas; HGG, high-grade gliomas; GI, grade I; GII, grade II; GIII, grade III; PPMS, primary progressive MS; SPMS, secondary progressive MS; RRMS, relapsing-remitting MS; WT, whole tumor; ET, enhanced tumor; TC, tumor core; WM, white matter; EDSS, Expanded Disability Status Scale.

Notably, various metrics are frequently selected for evaluation based on their prior use in reference papers for comparison with newly developed methods. While this approach facilitates direct comparisons, it also creates a cycle that reinforces the use of suboptimal metrics (Yeghiazaryan and Voiculescu, 2018).

It is important to note that while accuracy is often used to quantify classification performance, its application in medical image segmentation is discouraged due to class imbalance between regions of interest (ROIs) and background. Consequently, when selecting an evaluation metric, it is critical to consider how the metric is affected by factors such as outliers, small segments, complex boundaries, or poor segmentation quality (Lefkovits and Lefkovits, 2022). Accuracy, Dice score, and sensitivity are all metrics derived from a confusion matrix for binary segmentation, which accounts for the number of true positives (TP), false positives (FP), true negatives (TN), and false negatives (FN) (Nghi, 2023). Accuracy is the number of correct predictions, consisting of correct positive and negative predictions divided by the total number of predictions (Nghi, 2023):

$$A\,c\,c\,u\,r\,a\,c\,y=\frac{T\,P+T\,N}{T\,P+T\,N+F\,N+F\,P}.$$

Dice score represents the overlap between predicted segmentation and ground truth (Nghi, 2023):

$$D\,i\,c\,e=\frac{2\,T\,P}{2\,T\,P+F\,P+F\,N}.$$

Sensitivity is the number of true positive results divided by the number of all samples that should have been identified as positive (Nghi, 2023):

$$S\,e\,n\,s\,i\,t\,i\,v\,i\,t\,y=\frac{T\,P}{T\,P+F\,N}.$$

The selection of performance metrics for assessing model performance is predominantly determined by the specific clinical context of the problem (Reinke et al., 2021). Bias can occur in different aspects of an ML study, including data handling, model development, and performance evaluation of models (Faghani et al., 2022). Given the flexibility in selecting performance metrics, some researchers, such as Demirel et al. (2021) and Wang et al. (2023), adopt a comprehensive approach by evaluating multiple metrics, including Accuracy and AUC, to provide a more robust assessment of the performance of their model. Lu et al. (2019) employs both Accuracy and Kappa as performance metrics in their evaluation. However, certain challenges explicitly specify which performance metrics should be used, ensuring a standardized assessment approach. Among the noted limitations and biases, Accuracy as a performance metric can lead to significant biases, particularly in scenarios with severe data imbalance. In such cases, Accuracy may overestimate the model’s performance by favoring the majority class, thereby failing to adequately reflect the model’s true predictive capability for the minority class. Zhong et al. (2017) employed a nested cross-validation strategy for model optimization and evaluation. The inner cross-validation loop was used for tuning and optimizing the model’s parameters, while the outer loop provided an unbiased assessment of the model’s performance, ensuring a robust evaluation process.

For handling imbalanced data, performance metrics like the F1 score, receiver operating characteristic (ROC) curve, or precision-recall (PR) curve may provide a more comprehensive evaluation of model performance compared to Accuracy (Faghani et al., 2022). These metrics account for the disparity between classes and offer a clearer representation of the model’s ability to identify minority class instances. Ortiz-Ramón et al. (2020) used the ROC curve to assess performance, highlighting the balance between sensitivity and specificity. To mitigate bias and ensure reliable results, the author considered only one lesion per patient and employed a fivefold cross-validation approach, which was repeated 10 times.

We can conclude that the choice of performance metrics is critical in evaluating the effectiveness of the models, as it directly impacts the interpretation of the results. By selecting metrics that align with the specific task and data characteristics (Faghani et al., 2022), and by employing techniques to mitigate bias, such as those demonstrated by Zhong et al. (2017) and Ortiz-Ramón et al. (2020), researchers can enhance the robustness and reliability of their findings.

### 3.3 Assessment of study quality

We used the radiomics quality score (RQS) (Lambin et al., 2012) to select the articles that, from a radiomic point of view, had the most rigorous and complex approach. An RQS toward the upper limit is obtained if certain criteria are ticked, for example: image protocols are well documented or public protocol is used; validation is single- or multicentric; it was prepared a report on the cost-effectiveness of the clinical application; make code and data publicly available (Lambin et al., 2012).

Table 4 presents the score of each item and the total score for each study. The mean RQS of all studies was 13.7 (38.05%) points, ranging from 6 (16.67%) to 23 (63.89%) points. Only five studies scored equal to or above 18 points (50%).

**TABLE 4:** Radiomics quality score results.

Reference	1. Image proto-col quality	2. Multiple segmen-tations	3. Phan-tom study	4. Imaging at multiple time points	5. Feature reduc-tion/ adjust-ment for multiple testing	6. Multi-variable analysis with non-radio-mics features	7. Bio-logical corre-lates	8. Cut-off analysis	9. Discri-mination statis-tics	10. Calibra-tion statis-tics	11. Prospec-tive study	12. Valida-tion	13. Compari-son to gold stan-dard	14. Poten-tial clinical utility	15. Cost-effective-ness analysis	16. Open science and data	17. Total	18. RQS (%)
Score range	0–2	0–1	0–1	0–1	0–3	0–1	0–1	0–1	0–2	0–2	0–7	0–5	0–2	0–2	0–1	0–4	0–36	0–100
Ortiz-Ramón et al., 2019	1	1	0	1	3	1	0	0	2	1	7	2	2	2	0	0	23	63.89
Kumar et al., 2023	1	1	0	1	3	0	0	0	2	1	7	2	2	2	0	0	22	61.12
Jain et al., 2021	1	0	0	0	3	0	0	0	1	1	7	2	2	2	0	1	20	55.56
Zhong et al., 2017	1	1	0	1	3	0	0	0	2	1	7	2	0	0	0	0	18	50.00
Combès et al., 2021	1	1	0	1	0	0	0	1	0	0	7	2	2	2	0	1	18	50.00
Eshaghi et al., 2021	1	1	0	0	3	0	1	0	1	1	0	5	0	2	0	1	16	44.44
Lu et al., 2019	1	1	0	1	3	1	1	0	1	1	0	2	2	2	0	0	16	44.44
Ortiz-Ramón et al., 2020	1	1	0	0	3	0	0	1	2	1	0	2	2	2	0	0	15	41.67
Cuocolo et al., 2020	1	1	0	0	3	0	1	0	2	2	0	2	2	0	0	0	14	38.89
Wang et al., 2023	1	1	0	0	3	1	0	1	2	1	0	2	0	2	0	0	14	38.89
Fenneteau et al., 2021	1	1	0	1	0	0	0	0	1	1	0	3	2	2	0	1	13	36.12
Sarica and Seker, 2022	1	1	0	1	3	0	0	0	0	0	0	2	2	0	0	2	12	33.33
Ashtari et al., 2022	1	1	0	1	0	0	0	0	1	1	0	2	2	2	0	1	12	33.33
Zhou et al., 2022	1	1	0	0	3	0	0	0	1	1	0	2	0	2	0	0	11	30.56
Elahi et al., 2020	1	0	0	0	3	0	0	0	0	0	0	5	2	0	0	0	11	30.56
Dastmalchian et al., 2021	1	1	0	0	3	0	0	1	1	1	0	0	2	0	0	0	10	27.78
Demirel et al., 2021	1	1	0	0	3	0	0	0	2	0	0	2	0	0	0	0	9	25.00
Lefkovits and Lefkovits, 2022	1	1	0	0	0	0	0	0	0	0	0	2	2	0	0	1	7	19.44
Pardini et al., 2015	1	0	0	0	0	1	1	0	1	1	0	0	0	2	0	0	7	19.44
Friconnet, 2021	1	1	0	0	0	1	0	0	1	0	0	2	0	0	0	0	6	16.67

The article by Ortiz-Ramón et al. (2019) lead the RQS scoreboard with 23 points (63.89%). The authors sought to classify scans utilizing RF and SVM classifiers, employing both feature selection and non-feature selection methodologies. To improve generalization of the model and robustness against overfitting in small samples the RF model was designed to combine the results of a multitude of independent and decorrelated decision trees in the training process. For the SVM model, a linear kernel significantly outperformed the others. For evaluating the efficiency of the classification models, a fivefold cross-validation approach was used. The best result was obtained using SVM with linear kernel.

The second article after RQS ranking (22 points or 61.12%) is from Kumar et al. (2023). The novelty of their approach was exemplified by extracting radiomics features from a single cross-sectional image of the T2W MRI sequence. Five different machine learning classifiers were used in the test cohort. Before segmentation and ROI delineation, image pre-processing was performed using the Laplacian of Gaussian (LOG) bandpass filters to remove the background noise (Gaussian filter) and to enhance the tumor edges (Laplacian filter). Thirty-six first-order features were extracted using various spatially scaled filters, and six shape (topographic) features were extracted without applying filters. The recursive feature removal method was used to remove weak features. The article evaluates the performance of five classification models (RF, Support Vector, Gradient Boosting, Naive Bayes, and Ada-Boost) and from these the RF classifier performed the best.

With an RQS of 55.56%, Jain et al. (2021) ranked 3rd. They performed feature extraction by using Pyradiomics Python package. For data augmentation (i) scaling, (ii) rotation, and (iii) shear were used. For classification tasks, various ensemble learning classification algorithms, such as RFs, bagging-based ensemble classifiers, and gradient-boosted ensemble classifiers like XGBoost and AdaBoost, were explored. A novel texture analysis matrix, termed Decreasing Gray-Level Matrix (DGLM), was proposed in the study. It was observed that boosting ensemble learning classifiers, such as AdaBoost and XGBoost, outperformed bagging and randomized classifiers.

Fourth place tied with 5th place: Zhong et al. (2017) and Combès et al. (2021) with an RQS of 50%. Zhong et al. (2017) performed minimal manual editing to remove nonbrain tissues and fill the holes in the white matter that occurred due to lesions. Pre-processing data was done and included (i) slice timing, (ii) motion correction with reference to the mean volume, (iii) skull stripping, and (iv) band-pass filtering. Three sets of features were investigated for classification ability: Set 1, structural features only; Set 2, functional features only; and Set 3, concatenated structural and functional features. Structural features performed slightly better than functional features. A linear SVM was employed for all feature sets. Classification performance was tested using leave-one-out cross-validation.

The other paper scored 50%, Combès et al. (2021) developed a complete workflow to facilitate the monitoring of new lesions on longitudinal MRI of MS patients. The workflow consists of three main components: (i) a software component that allows for automated and secured anonymization and transfer of MRI data, (ii) a fully automated segmentation core that enables detection of focal longitudinal changes in patients, and (iii) a dedicated web viewer that provides an intuitive visualization of new lesions to radiologists and neurologists. A 3D U-Net model employing 6 input channels was utilized for segmentation. Data post-processing consisted of first a softmax outputs map, second connected components extracted from the resulting binary map and third only connected components with volume >12 mm^3^ were considered.

### 3.4 Segmentation of new MS lesions

Out of the 20 articles eligible for this review, 5 aimed to segment and detect new lesions (Elahi et al., 2020; Combès et al., 2021; Fenneteau et al., 2021; Ashtari et al., 2022; Sarica and Seker, 2022). They evaluated the performance by computing Sensitivity (Elahi et al., 2020; Combès et al., 2021) and Dice scores (Fenneteau et al., 2021; Ashtari et al., 2022; Sarica and Seker, 2022). An impediment to compare the performances obtained by different authors is that different research teams use different metrics. The best sensitivity was obtained by Combès et al. (2021) (90%). They analyzed T1W, T2W, and T2-FLAIR images, and proposed the following workflow: a software to automatically, securely, and anonymously transmit the images to a server where they were processed, a fully automatic lesion segmentation system and a web application dedicated to visualizing the lesions. This article used a 3D U-Net model with six input channels (for each sequence and each time point). The preprocessing operations were: (I) volumes were reoriented in RAS coordinates, (II) skull and skin tissues were removed from the data, and (III) bias due to spatial inhomogeneity was estimated using the N4 algorithm and removed from the data. For data augmentation (i) isotropic re-scaling, (ii) 3D rotation, (iii) mirroring in the sagittal plane, (iv) smooth elastic deformations, and (v) intensity enhancements were used. The performance of the proposed workflow was evaluated by comparing the maximum sensitivity obtained by three neuroradiological experts, working without the workflow (0.74) and the sensitivity (0.9) with the workflow.

Fenneteau et al. (2021) obtained the highest score (65%). They used an MPU-net (Minimally Parameterized U-net) type model, which uses a small number of parameters, proving that it is possible to learn a performant model with only 10 fully annotated examples. Nine variations of the MPU-net architecture were analyzed. Adding batch normalization, dropout layers and including residual blocks in the encoder part of the model. For the MPU-net++ template, the number of consecutive convolutions in each block was also evaluated.

## 4 Discussion

By implementing successive filtering based on exclusion criteria (such as non-English versions, non-MRI techniques, and exclusion of animal studies) 20 articles were identified and included in this review. Following the calculation of the RQS, only five articles achieved a score exceeding 50%. All five of these articles were prospective studies, which inherently awarded them an additional 7 points, resulting in a relative increase of 19.44% in their scores. Given the influence of study design on scoring, we conducted a separate evaluation for retrospective studies, revealing that three articles in this category scored above 40%. It is essential to recognize that a lower RQS does not necessarily imply a lack of scientific validity. One straightforward method to enhance an article’s RQS (by up to 4 points or 11.11%) is to provide open-source access to the underlying code, ROIs, radiomic features, or the imaging data used in the study. This approach promotes transparency and reproducibility, which are critical elements in radiomics research.

The data in Table 3 show that there is no consensus on the model used to segment or classify MS lesions. For classification the established models are used, e.g., U-net for segmentation (Combès et al., 2021; Fenneteau et al., 2021; Sarica and Seker, 2022) and SVM, RF, or *K*-Nearest Neighbors (Zhong et al., 2017; Ortiz-Ramón et al., 2020; Wang et al., 2023). Nonetheless, sometimes different solutions are also used: e.g., Subtype and Staging Inference (Eshaghi et al., 2021) or a Meta-classifier (Elahi et al., 2020).

Regarding the evaluation of the performance of the models, there is no unanimous criterion used. For segmentation of lesions, the authors used standard metrics to evaluate medical image segmentation performance: Accuracy, Dice score, and Sensitivity.

We analyzed the articles that obtained the best result according to Accuracy, Sensitivity, and Dice score (see Table 3) with the aim to identify the differences between these studies and to determine the factors contributing to their results.

To enhance accuracy, Jain et al. (2021) applied data augmentation and feature selection, combined with gradient-boosted ensemble learning classifiers, to improve model performance and increase classification accuracy. The model’s performance can be enhanced by incorporating external datasets.

Combès et al. (2021) achieved the best segmentation performance, with a sensitivity of 0.9. They used longitudinal MRI data, integrating radiomic features with deep learning models. They employed an open-source nnU-Net model with six input channels, one for each MRI sequence and timepoint. Each input image was first resampled, and then each set of six images was divided into patches of a specified size. Finally, each such six patches were processed independently and aggregated to others to form the final softmax output map. However, the results must be interpreted in the context of the specific population used in the study. In addition, all FLAIR, T2-w, and T1-w images were used as input to the automatic lesion detection segmentation module, therefore, if not all three sequences are available, it needs to be adjusted.

Lefkovits and Lefkovits (2022) achieved the highest Dice score, evaluating the performance of the U-Net model, VGG16-UNet, and ResNet-UNet, respectively. They concentrated on both preprocessing and post-processing steps to enhance detection performance. The intensity of the original images was adjusted, correcting the images and transforming the varying intensity ranges into the 8-bit grayscale domain to standardize tissue intensities while maintaining the original histogram shapes. In addition, they demonstrated that combining the three models into an ensemble model resulted in an overall performance improvement of 2%.

For a comprehensive analysis, we have detailed the strengths and limitations of each article included in the review to establish a guiding framework. This summary is presented in Table 5.

**TABLE 5:** Strengths and limitations of the included articles.

Reference	Strengths	Limitations
Ortiz-Ramón et al., 2019	Large dataset 1,800 3D MRI Radiomics-based approach Wide range of features Feature selection techniques	Potential overlap in MRI signal intensities Lack of clear influence of feature selection Minimal comparison with state-of-the-art
Kumar et al., 2023	Radiomic texture analysis combined with machine learning A diverse dataset of MRI Feature extraction	Dataset imbalance Retrospective study
Jain et al., 2021	Ensemble learning classifiers Radiomics-based approach Feature selection techniques Open data access	No cross-validation
Zhong et al., 2017	Combines structural and functional MRI data Data-driven feature extraction Multivariate approaches	Data at a single point in time Small sample size
Combès et al., 2021	Integration with clinical workflow longitudinal MRI data Radiomic features and deep learning models External validation and clinical testing	Dependence on MRI quality and protocol standardization Need all FLAIR, T2-w, and T1-w images as input
Eshaghi et al., 2021	Unsupervised machine learning methods Multi-modal MRI data Dimensionality reduction techniques such as principal component analysis (PCA) to compress high-dimensional MRI data Longitudinal data and temporal analysis Cross-validation	The model’s performance could be population-specific Without a clear ground truth
Lu et al., 2019	Cross-validation Radiomic texture analysis combined with machine learning	Feature redundancy Need validated across external datasets
Ortiz-Ramón et al., 2020	Multi-parametric MRI sequences External validation using independent datasets from different institutions	Class imbalance
Cuocolo et al., 2020	Radiomic texture analysis combined with machine learning Recursive feature elimination (RFE) Extensive number of texture features Hyperparameter tuning via cross-validation SMOTE (Synthetic Minority Oversampling Technique) to balance the dataset	Small sample size Manual segmentation Data from a single institution Only T2-weighted MRI data
Wang et al., 2023	Radiomic texture analysis combined with machine learning Feature selection techniques	Sample size and class imbalance Need validated across external datasets
Fenneteau et al., 2021	Minimum parameters U-Net Transfer learning function	No texture analysis
Sarica and Seker, 2022	Attention U-Net and residual U-Net Utilization of 2D slices from 3D MR images Open data access	Need validated across external datasets Should employ cross-validation
Ashtari et al., 2022	U-Net model, incorporating pre-activation layers (batch normalization and activation functions (such as ReLU) are applied before the convolutional layers)	Sensitivity to data preprocessing
Zhou et al., 2022	Radiomic texture analysis combined with machine learning Enhanced T1-weighted MRI Involvement of multiple institutions	Need validated across external datasets Limited methodology details
Elahi et al., 2020	Multi-center data for improved generalizability Texture classification and texture features Cross-validation	Dependence on preprocessing steps Need for larger datasets Computational complexity
Dastmalchian et al., 2021	Radiomic texture analysis combined with machine learning Feature selection techniques	Small sample size Single slice used not 3D
Demirel et al., 2021	Automatic segmentation Radiomic texture analysis combined with machine learning	Lack of specific methodology details Potential for overfitting Dataset with a limited number of cases
Lefkovits and Lefkovits, 2022	Exploration of U-Net variants (e.g., attention U-Net and residual U-Net) Cross-validation	Dependence on histogram correction Need validated across external datasets
Pardini et al., 2015	Correlation with clinical disability Multimodal imaging approach	Lack of specific algorithm details Insufficient cross-validation or external validation
Friconnet, 2021	Correlating texture features with semantic descriptors (e.g., repetitiveness and roughness) 300 brain MRI scans with various imaging modalities (T1-, T2-, and diffusion-weighted MRI) Good inter-rater reliability	Limited results transferability Just 32 texture features used

We have pinpointed several key recommendations for enhancing reproducibility and achieving a high RQS score: (i) use open-source datasets, such as publicly available image repositories, and to offer a thorough description or access to the model employed. This practice helps to avoid a “black box” scenario, where only the inputs and outputs are provided, and ensures greater transparency and clarity in the research methodology (ii) combine radiomics with machine learning models, it is well-established that integrating both techniques yields superior results compared to using machine learning models alone (iii) employ feature selection techniques to prevent feature redundancy and retain only the most relevant features (iv) addressing the data imbalance in the dataset with data augmentation techniques, such as the Synthetic Minority Oversampling Technique (SMOTE), could be a viable solution (Cuocolo et al., 2020) (v) to confirm the model’s generalizability and performance in other settings is necessary an external validation on independent datasets (vi) use of multi-modal MRI data, which includes structural, diffusion, and functional MRI sequences.

These diverse imaging modalities provide a comprehensive view of both macrostructural and microstructural changes in MS, improving the model’s ability to identify meaningful patterns and differentiate between subtypes. One could refer to dimensionality reduction techniques such as principal component analysis (PCA) or t-distributed Stochastic Neighbor Embedding (t-SNE) to compress high-dimensional MRI data into a lower-dimensional space that can be more easily interpreted by machine learning models. This approach reduces noise and computational complexity while preserving critical features, allowing for efficient clustering of MS subtypes.

Jain et al. (2021) and Lefkovits and Lefkovits (2022) who achieved the highest Accuracy and Dice score, respectively, opted for an ensemble model. This approach combines the predictive strengths of multiple machine learning models to enhance accuracy and robustness.

Although it was not the primary focus of this review article, we have included insights and ideas from relevant studies conducted on CT images, as well as those involving animal models. These investigations may provide innovative approaches that could be applied to the machine learning algorithms employed for analyzing MRI data from human participants. By examining these parallels, we can identify potential advancements that could enhance the efficacy of the MRI-based diagnostic methods.

To provide a broader perspective, we have incorporated observations from related studies that employed machine learning algorithms on CT images, as referenced in sources (Lee and Fujita, 2020; Salehinejad et al., 2021; Chen et al., 2022). These studies offer valuable insights that complement our findings and help contextualize the application of ML techniques in different imaging modalities. In CT image analysis, general features such as shape, pixel intensity, location, and statistical texture are commonly examined. Texture features are often based on the co-occurrence matrix (Haralick et al., 1973) and gray-level difference statistics (Weszka et al., 1976), providing detailed insights into the patterns and relationships within the image data.

Among the machine learning models employed, notable mentions include CNN-2, VGG-16, ResNet-50 (Chen et al., 2022), SE-ResNeXt-50 (Salehinejad et al., 2021), and AlexNet (Lee and Fujita, 2020). Like the machine learning algorithms applied in MRI, those used for CT can focus on either segmenting regions [e.g., Lee and Fujita (2020), where the input for segmentation is a 3D CT image, resulting in a label map that annotates anatomical structures with predefined labels] or on binary classification [as demonstrated by Salehinejad et al. (2021)]. Additionally, some models are designed for multiclass classification, such as those proposed by Lee and Fujita (2020).

In related studies conducted on animal models (Spiteri et al., 2019; Biercher et al., 2021; Zheng et al., 2022), it was observed that the methodologies closely mirror those employed in human subjects, particularly regarding image pre-processing and the application of texture analysis. A comprehensive set of parameters is typically extracted, which are then systematically analyzed and organized using statistical methods, even though these parameters may not always lend themselves to straightforward interpretation. Techniques such as SVM and RF (Spiteri et al., 2019) are utilized for classification tasks, such as differentiating between control and diseased groups in animal studies (e.g., two cohorts of dogs). Additionally, convolutional neural networks (CNNs) have been applied to detect the presence or absence of lesions in canine MRI scans (Biercher et al., 2021). While there may be unique elements in the workflows or models specific to animal research, these distinctions were not explicitly identified in the reviewed studies.

Within the domain of decision support tools in MS diagnosis, it is essential to converge on a consensus regarding both the selection of the model employed and the establishment of standardized performance metrics for its robust evaluation. Thus, to encourage the development of robust solutions and move the community away from small-scale image classification tasks and toward realistic, complex tasks taken from real-world challenges which provides a dataset with preprocessed and annotated images [e.g., Shifts Challenge (2022a) and Commowick et al. (2021a)]. Such challenges represent a benchmark and a starting point for the development of segmentation solutions and automatic detection of MS lesions.

Several key challenges have arisen over the past 16 years, starting at the Medical Image Computing and Computer Assisted Intervention (MICCAI) conference in 2008 (Styner et al., 2008). The database of this challenge included 20 training sets (10 from scanner 1 and 10 from scanner 2 all with manual segmentations) and 25 testing sets (15 from scanner 1 and 10 from scanner 2 without expert segmentations). A second test set was included to prevent overfitting. The rationale for using two separate test sets was that distributing test data alongside the training data allows teams to fine-tune their algorithms for the known test cases. The full testing database was segmented by a single expert rater at CHB and independently by two expert raters at UNC, resulting in two comprehensive sets of expert segmentations that served as references for comparison. For all cases, the database contained the same number of high-resolution images: a T1 weighted scan, a T2 weighted image, a FLAIR image, a diffusion tensor imaging (DTI) derived fractional anisotropy (FA) and mean diffusivity (MD) image. To evaluate the quality of automatic segmentation the following four error metrics were used: Relative absolute volume difference (the total absolute volume difference of the segmentation to the reference divided by the total volume of the reference, in percent), Average symmetric surface distance, in millimeters (to analyze the border voxels of segmentation and reference border), True Positive Rate and False Positive Rate. Approximately one-third of teams submitted results, with many of them achieving scores within a similar range of variability typically seen among different human raters.

Another major challenge took place at the IEEE ISBI international conference in 2015 (Carass et al., 2017). The organizers provided 82 datasets from a single 3.0 Tesla MRI scanner, each containing an average of 4.4 time-points. All images had their lesions manually delineated in the MNI space by two raters. To mitigate potential biases from relying solely on individual raters, the organizers opted to create a Consensus Delineation for the images using the Simultaneous Truth and Performance Level Estimation (STAPLE) algorithm (Warfield et al., 2004). This method allows for a more balanced and reliable delineation by combining the segmentations from both raters to generate a consensus label. In brief, STAPLE estimates the true segmentation from an optimal combination of the input segmentations, the weights for which are determined by the estimated performance level of the individual segmentations. To compare the results from the participants with the two manual raters and Consensus Delineation, the organizers used the following metrics: Dice overlap, positive predictive value, true positive rate, lesion true positive rate, lesion false positive rate, absolute volume difference, average symmetric surface distance, volume correlation, and longitudinal volume correlation. Ten teams participated in the challenge, where seven teams used supervised algorithms and three employed unsupervised algorithms. The best algorithm depends on the criteria used for evaluation. As for Longitudinal Correlation the IIT Madras team stands out, using two CNNs, each trained on data from one rater, and their outputs were combined for the final segmentation. However, for Dice overlap, Team PVG One achieved the best result using a Hierarchical MRF and Random Forest Segmentation.

A valuable open-source dataset was provided by the MICCAI 2016 challenge (Commowick et al., 2021b), which included 53 image datasets, annotated by 7 manual delineations from expert raters. Hyperintense lesions on FLAIR were manually delineated on each patient with control on T2 sequence and gathered in a consensus segmentation for evaluation. The purpose of this dataset was to become a reference in MS lesions segmentation evaluation. The challenge revealed a significant limitation: it was not feasible to evaluate computational performance (e.g., memory usage or processing time). This highlights the need for centralized computing platforms that support challenges by offering data storage, processing pipeline integration, and evaluation workflows on shared datasets. Such platforms would allow for a fair comparison among fully automated methods. Additionally, segmentation challenges face issues with ground truth accuracy due to limited manual delineations. In addition to the individual automatic algorithms, the challenge organizers created a composite model named “team fusion.” This approach combined segmentations from all 13 teams participating in the challenge, using label fusion through the LOP STAPLE algorithm to reach a consensus segmentation. This method aimed to integrate insights from various approaches, producing a segmentation that leveraged the strengths of multiple algorithms for improved accuracy. The goal of this fourteenth method was to evaluate the capability of such a label fusion method to overcome the individual difficulties of each method and thus obtain results closer to the ground truth. This composite algorithm improved the average results of individual automatic algorithms for all metrics, suggesting its ability to incorporate the best solutions into a consensus segmentation.

A worthful publicly available dataset was developed by Lesjak et al. (2018) consisting of MRI data from 30 patients diagnosed with MS. This dataset includes a unique protocol designed to generate reference segmentations of white-matter lesions based on a multi-rater consensus approach, enhancing the reliability and consistency of lesion annotations. In addition to providing open-source access to the dataset, the authors provided a comprehensive documentation, which enhanced the dataset’s utility for other researchers and facilitates reproducibility in studies involving MS lesion analysis. Initially, each rater independently segmented the lesions on all 30 datasets. For this purpose, raters used the BrainSeg3D’s semi-automated tool. In the following, they had several joint sessions to create the validated consensus segmentation of the lesions.

Another recent example is the Shifts Challenge from 2022 (Shifts Challenge (2022a)) which serves as pivotal benchmarks and initial steps in the advancement of segmentation solutions and automated detection methodologies for MS lesions. This challenge also establishes the performance matrix used to evaluate participants’ performance, namely the lowest area under the error retention curve (R-AUC) in this case. There were 46 solutions submitted, and the best solution obtained an R-AUC 0.0128 ± 0.0169 by the team led by Adrián Galdrán (Shifts Challenge (2022b)).

An additional relevant challenge is the BraTS 2023 Intracranial Meningioma Segmentation Challenge, which marks a significant advance in tumor segmentation while highlighting the potential and limitations of current methods (LaBella et al., 2024). The BraTS Meningioma Challenge received image data from six U.S. academic medical centers: Yale University, Missouri University, Thomas Jefferson University, Duke University, University of California, San Francisco, and University of Pennsylvania. This dataset comprised T1, T2, FLAIR, and T1Gd brain MRI sequences from patients diagnosed with intracranial meningiomas. Nine teams developed deep-learning segmentation models using the largest multi-institutional, expert-annotated meningioma MRI dataset. Metrics used for evaluation included Dice Similarity Coefficient (Nghi, 2023) and the 95% Hausdorff Distance (95HD) (Rucklidge(ed.), 1996) and were evaluated on a lesion-wise level. The Hausdorff Distance metric was chosen since it measures the degree of mismatch between two sets by finding the point of A that is farthest from any point of B and vice versa. The NVAUTO team lead by Andriy Myronenko achieved the highest scores with DSC of 0.904 ± 0.180 and a 95HD of 31.4 ± 71.8. They developed an algorithm named as AutoSeg3D, an open-source framework based on PyTorch, which is particularly adaptable to various automated segmentation challenges in medical imaging (LaBella et al., 2024). Auto3DSeg supports auto-scaling to available GPUs, enables fivefold training with SegResNet, DiNTS, and SwinUNETR models, and facilitates inference and ensembling using each of the multiple trained models (LaBella et al., 2024).

To summarize:

1. Our search using the specified keywords initially yielded 1,157 articles from WOS and Scopus databases. Through successive filtering based on the predefined criteria, we narrowed down the selection to 20 articles, which constituted the focus of this review. Notably, the selected articles span the interval from 2015 to 2023. Using the RQS, we meticulously assessed articles to discern those with the most methodological rigor and complexity in the realm of radiomics. Following computation of RQS scores, merely five articles surpassed the 50% threshold. Noteworthy, all five articles were prospective studies. Moreover, a distinct evaluation of retrospective articles identified three entries with RQS scores exceeding 40%.
2. Model variability: There is no consensus on the models used for segmenting and classifying MS lesions. Common segmentation models include U-net, while classifiers like SVM, RF, and *K*-Nearest Neighbors are popular. More unique approaches, such as Subtype and Staging Inference and Meta-classifiers, were also noted.
3. Performance metrics: Standard metrics (Accuracy, Dice score, and Sensitivity) were used for segmentation evaluations. Jain et al. (2021) improved accuracy through data augmentation and external datasets, while Combès et al. (2021) achieved high sensitivity (0.9) by incorporating longitudinal MRI data and radiomic features. Lefkovits and Lefkovits (2022) attained the best Dice score by standardizing image intensities and using ensemble models.
4. Key recommendations: Suggestions to enhance reproducibility include using open-source datasets, combining radiomics with machine learning, addressing data imbalance through augmentation, and ensuring external validation. The use of multi-modal MRI data is recommended for a more comprehensive analysis of MS lesions.

1. Challenges and benchmarks: The importance of standardized models and evaluation metrics in MS diagnosis is emphasized. Recent challenges like the Shifts Challenge 2022 and BraTS 2023 provide crucial benchmarks for advancing segmentation and detection methods.

## 5 Conclusion

Consensus on the model used for segmenting or classifying MS lesions is currently lacking, with prevalent models such as U-net, SVM, RF, and *K*-Nearest Neighbors. Additionally, other segmentation and classification methods as Subtype and Staging Inference or a Meta-classifier are also explored. Evaluation of performance also lacks consensus, with metrics like Accuracy, Dice score, and Sensitivity commonly used in assessing lesion segmentation efficacy in medical imaging.

In conclusion, our article provides an insight into the contemporary landscape of decision support tools that leverage texture analysis and artificial intelligence for the analysis and monitoring of emerging MS lesions in MRI images.

## Data Availability

The raw data supporting the conclusions of this article will be made available by the authors, without undue reservation.
